# Mining of Indian wheat germplasm collection for adult plant resistance to leaf rust

**DOI:** 10.1371/journal.pone.0213468

**Published:** 2019-03-28

**Authors:** Sundeep Kumar, B. S. Phogat, V. K. Vikas, A. K. Sharma, M. S. Saharan, Amit Kumar Singh, Jyoti Kumari, Rakesh Singh, Sherry Rachel Jacob, G. P. Singh, M. Sivasamy, P. Jayaprakash, M. Meeta, J. P. Jaiswal, Deep Shikha, B. K. Honrao, I. K. Kalappanavar, P. C. Mishra, S. P. Singh, S. S. Vaish, V. A. Solanki

**Affiliations:** 1 ICAR-National Bureau of Plant Genetic Resources, Pusa Campus, New Delhi, India; 2 ICAR-Indian Agricultural Research Institute, Regional Station, Wellington, Tamilnadu, India; 3 ICAR-Indian Institute of Wheat & Barley Research, Karnal, India; 4 Punjab Agricultural University, Ludhiana, Punjab, India; 5 Govind Ballabh Pant University of Agriculture and Technology, Pantnagar, Uttarakhand, India; 6 Agharkar Research Institute, Pune, Maharashtra, India; 7 University of Agricultural Sciences, Dharwad, Karnataka, India; 8 Zonal Agricultural Research Station (JNKVV), Powarkheda, Madhya Pradesh, India; 9 Narendra Deva University of Agriculture and Technology, Faizabad, Uttar Pradesh, India; 10 Banaras Hindu University, Varanasi, Uttar Pradesh, India; 11 Sardarkrushinagar Dantiwada Agricultural University, Vijapur, Gujarat, India; Mahatma Phule Krishi Vidyapeeth College of Agriculture, INDIA

## Abstract

Leaf rust (*Puccinia triticina* Eriks.) is a fungal disease of wheat (*Triticum* spp.), which causes considerable yield loss. Adult plant resistance (APR) is one of the most sustainable approaches to control leaf rust. In this study, field-testing was carried out across ten different locations, followed by molecular screening, to detect the presence of APR genes, *Lr34*+, *Lr46*+, *Lr67*+ and *Lr68* in Indian wheat germplasm. In field screening, 190 wheat accessions were selected from 6,319 accessions based on leaf tip necrosis (LTN), disease severity and the average coefficient of infection. Molecular screening revealed that 73% of the accessions possessed known APR genes either as single or as a combination of two or three genes. The occurrence of increased LTN intensity, decreased leaf rust severity and greater expression of APR genes were more in relatively cooler locations. In 52 lines, although the presence of the APR genes was not detected, it still displayed high levels of resistance. Furthermore, 49 accessions possessing either two or three APR genes were evaluated for stability across locations for grain yield. It emerged that eight accessions had wider adaptability. Resistance based on APR genes, in the background of high yielding cultivars, is expected to provide a high level of race non-specific resistance, which is durable.

## Introduction

Wheat is one of the most important field crops in the world because it provides 20% of the total energy and protein in the human diet [1], thus playing a crucial role in global food security. Global wheat yield has reached 736 mt [2], but the demand for wheat continues to increase due to the ever-growing world population, which is expected to reach 9.7 billion by 2050 [3]. In India, demand for wheat is estimated to be ~109 million tonnes in 2020 [4]. However, in the presence of a number of biotic stresses like rust (leaf, stem and stripe) and diseases like wheat blast, which are emerging as new threats due to climate change, this target will be difficult to achieve. Leaf rust caused by *Puccinia triticina* Eriks (previously known as *Puccinia recondita* f. sp. *tritici*) is deemed to be a major disease in almost all parts of the world where wheat is grown [5], and it causes significant yield loss. The average yield loss due to leaf rust (brown rust) varies from 15% to 60% [6]. However, the extent of yield reduction depends on the stage of plant growth at which infection occurs, the degree of resistance of cultivars and disease severity, which, in turn, depends on weather conditions [7].

Use of resistant cultivars is the most economic, reliable and environmentally safe way to control rust diseases [8]. Historically, in wheat many genes were deployed to provide resistance against these diseases. However, most of the rust resistance genes are race-specific and controlled by one or few major genes, which are often short lived due to the ability of the pathogen to become virulent after single-step mutation. There are examples where enhanced resistance have been achieved through pyramiding of major (race-specific) resistance genes, however this alone is not sufficient to provide a sustainable level of resistance. Over 70 leaf rust resistance genes have been identified and incorporated in the agronomically superior lines, but due to a higher rate of breakdown of resistance genes and their narrow genetic base, it is difficult to achieve durable resistance in the breeding material [9]. Alternatively, incorporating major resistance genes with non-specific resistance (slow rusting) can provide durable disease resistance [10].

Adult plant resistance (APR) genes are known to confer partial resistance in a non-specific way to one or more rust diseases. APR is best expressed at the adult plant stage wherein resistance is conferred by multiple additive genes possessing quantitative inheritance. Since APR is generally governed by multiple additive genes, it is not subjected to the “boom and bust cycle” of disease epidemics. Therefore, APR is considered more durable than all stage resistance (ASR) or seedling resistance or race-specific resistance, which is governed by a major gene providing hypersensitive response (HR). Generally, APR is considered non-race-specific, but there are exceptions where some genes provide race-specific resistance such as *Lr13* [11], while some confer HR response like *Lr48* [12].

The effect of individual APR genes was found to be partial and unsatisfactory under high disease pressure; however, combining them with other APR genes was able to confer high levels of adult plant resistance [13]. Among the catalogued rust resistance genes, leaf rust resistance genes, *Lr34/Yr18/Sr57/Pm38/Ltn1* (hereafter referred as *Lr34*+), *Lr46/Yr29/Sr58/Pm39/Ltn2* (hereafter referred as *Lr46*+), *Lr67/Yr46/Sr55/Pm46/Ltn3* (hereafter referred as *Lr67*+) and *Lr68* are APR genes which have been widely used for imparting durable resistance. APR genes *Lr34*+, *Lr46*+, *Lr67*+ are multipathogen effective and provide broad spectrum resistance to leaf rust, stripe rust, stem rust and powdery mildew, i.e. *Lr34/Yr18/Sr57/Pm38/Ltn1*, *Lr46/Yr29/Sr58/Pm39/Ltn2* and *Lr67/Yr46/Sr55/Pm46/Ltn3*, respectively. APR genes are also associated with leaf tip necrosis (LTN), a phenotypical marker that is linked or pleiotropic with these genes [14]. LTN appears on the flag leaves when plants are grown in fields [15, 16]. LTN is one of the important traits for selecting genotypes with APR genes in conventional wheat breeding programs. However, this trait is sensitive to environmental factors and employing molecular markers provides a reliable option to confirm the presence of APR genes.

In the last two decades, advances in the field of molecular markers have contributed significantly to the identification of genes for resistance to various leaf rust races and utilization of these genes in marker assisted selection (MAS) [17]. Indigenous wheat germplasm accessions conserved in the National Genebank have not been evaluated for adult plant leaf rust disease resistance in the past, either at phenotypic or genotypic level. Characterization of the germplasm for specific trait like leaf rust reaction imparts value to it. Conservation of germplasm in the genebank becomes meaningful only if its diversity is exploited efficiently. With this background information, the present investigation was carried out to identify new and diverse sources of resistance for leaf rust in Indian wheat germplasm. Our study characterized 6,319 Indian wheat germplasm lines for APR genes, initially through phenotypical marker (LTN), disease severity and average coefficient of infection (ACI). The phenotypically confirmed resistance lines were further subjected to molecular marker analyses for the presence or absence of known APR genes such as *Lr34*+, *Lr46*+, *Lr67*+ and *Lr68*. Promising lines with two and three APR gene combinations were also studied for stability of yield using additive main effect and multiplicative interaction (AMMI) analysis.

## Materials and methods

Initially a set of 9,630 indigenous wheat germplasm accessions conserved in National Genebank at ICAR-National Bureau of Plant Genetic Resources (NBPGR), New Delhi were multiplied. For purification of line, single plant from each of the accessions were grown in the field and further subjected to a generation of single seed descent (SSD) to develop genetically stable lines. These stable lines were characterized using wheat descriptors (30 traits) to eliminate duplicates based on the phenotypic traits. Finally, 6,319 indigenous wheat germplasm lines were selected for characterization to evaluate adult plant resistance.

### Screening of wheat germplasm for the presence of LTN trait and leaf rust reaction

Germplasm lines were screened under field conditions and using artificial inoculation for the presence of LTN, a phenotypical trait linked to the APR genes, along with leaf rust reaction, at ten locations in India for two years. The locations were Pantnagar (Uttarakhand), Ludhiana (Punjab), Karnal (Haryana), Varanasi (Uttar Pradesh), Kumarganj (Uttar Pradesh), Vijapur (Gujarat), Powarkheda (Madhya Pradesh), Pune (Maharashtra), Dharwad (Karnataka) and Wellington (Tamilnadu). Together, they represent most of the agro-climatic zones of wheat cultivation in India. Pantnagar, Ludhiana, Karnal, Varanasi, Kumarganj and Wellington have relatively cooler weather than Vijapur, Powarkheda, Pune and Dharwad during the growing season. All the lines were planted in one-meter rows with a space of 23 cm between the rows at all locations. Three spreader rows (row-to-row gap of 15 cm) of a mixture of susceptible cultivars (WL 711, Lal Bahadur, C 306, Agra Local and Karchia Local) were planted on four sides of the plot and one row of spreader was planted in between the test material after every 20 rows. All the recommended agronomic practices were followed throughout the crop duration. Based on the reports of Juliana et al [18], LTN was categorized as low, medium, high and very high.

Leaf rust epidemic was initiated by inoculating 3 week-old plants of spreaders, with urediniospore-water-tween 20 suspension having equal proportions of predominant leaf rust pathotypes of the particular location. Response of wheat germplasm to rust severity was recorded following the modified Cobb scale [19] after the accessions had completed the growth stage 87 [20]. Disease scores were determined by taking into account the severity of disease on plant parts, recorded as percentage of area covered (5%, 10%, 20%, 40%, 60%, 80% and 100%) and kind of host response as described by Loegering [21] (0- No visible infection, R- Resistant: necrotic areas with or without minute uredia, MR- Moderately resistant: small uredia, surrounded by necrotic areas, MX- Intermediate: variable sized uredia, some with necrosis or chlorosis, MS- Moderately susceptible: medium uredia with no chlorosis present, S- Susceptible: large uredia, no necrosis or chlorosis). Quantification of rust resistance was calculated using coefficient of infection (CI) values obtained by multiplying the disease severity with the standard values of host response (Pustule type) as per Pathan and Park [22]. Once the CI of a particular location was calculated, average coefficient of infection (ACI) based on multi-location data was computed. ACI was calculated based on data for 10 locations. ACI values of 0–20, 21–40 and 41–60 were regarded as possessing high, moderate and low levels of APR, respectively. The highest rust score amongst the locations was taken as the rust reaction of the accessions. An accession is considered resistant in the scenario where ACI is ≤ 20.00 and rust reaction is ≤ 20S. Near isogenic lines in the Avocet background viz., Avocet+*Lr34*, Avocet+*Lr46* and Avocet+*Lr67* were also included as the control.

Leaf rust pathotypes prevalent in the tested locations viz., 77–1 (109R63), 77-5(121R63-1), 77-9(121R60-1), 104-1(21R31-1), 104-2(21R55) and 104-3(21R63) were used for the study. Not every pathotype was present in all locations; each location had 2 or 3 predominant pathotypes among the listed pathotypes. Avirulence/virulence profiles of the pathotypes are documented in Table 1. Mixtures of predominant pathotypes of the respective location were used for artificial inoculation.

**Table 1:** Avirulent and virulent profile of pathotypes prevalent in the tested locations.

Sl. No.	Pathotypes	Avirulence on genes	Virulence on genes
1	77–1 (109R63)	*Lr9*, *19*, *24*, *25*, *26*, *28*, *29*, *32*, *36*, *39*, *42*, *43*, *44*, *45*, *47*	*Lr1*,*2a*, *2b*, *2c*, *3*,*10*, *11*, *12*,*13*, *14a*, *14b*, *15*, *16*, *17a*, *17b*, *18*, *20*, *21*, *22a*, *22b*, *23*, *27*, *31*, *30*, *33*, *35*, *37*, *38*, *40*, *48* and *49*
2	77-5(121R63-1)	*Lr9*, *19*, *24*, *25*, *28*, *29*, *32*, *39*, *42*, *43*, *45*, *47*	*Lr1*,*2a*, *2b*, *2c*, *3*,*10*, *11*, *12*,*13*, *14a*, *14b*, *15*, *16*, *17a*, *17b*, *18*, *20*, *21*, *22a*, *22b*, *23*, *26*, *27*, *30*, *33*, *35*, *36*, *37*, *38*, *40*, *44*,*48* and *49*
3	77-9(121R60-1)	*Lr2a*, *2b*, *2c*, *9*, *19*, *24*, *25*, *28*, *32*, *39*, *42*, *45*, *47*	*Lr1*, *3*, *10*, *11*, *12*, *13*, *14a*, *14b*, *15*, *16*, *17a*, *17b*, *18*, *20*, *21*, *22a*, *22b*, *23*, *26*, *27+31*, *30*, *33*, *35*, *36*, *37*, *38*, *44*, *46*, *48* and *49*
4	104-1(21R31-1)	*Lr2a*, *9*, *15*, *19*, *24*, *25*, *26*, *28*, *29*, *32*, *39*, *42*, *43*, *45*, *47*	*Lr1*, *2b*, *2c*, *3*, *10*, *11*, *12*, *13*, *14a*, *14b*, *16*, *17a*, *17b*, *18*, *20*, *21*, *22a*, *22b*, *23*, *27*, *30*, *33*, *35*, *36*, *37*, *38*, *40*, *44*, *48* and *49*
5	104-2(21R55)	*Lr9*, *10*, *13*, *15*, *19*, *20*, *24*, *25*, *28*, *29*, *32*, *36*, *39*, *42*, *43*, *45*, *47*	*Lr1*,*2a*, *2b*, *2c*, *3*, *11*,*12*, *14a*, *14b*, *16*, *17a*, *17b*, *18*, *21*, *22a*, *22b*, *23*, *26*, *27+31*, *30*, *33*, *34*, *35*, *37*, *38*, *40*, *44*, *48* and *49*
6	104-3(21R63)	*Lr9*, *10*, *13*, *15*, *17a*, *19*, *24*, *25*, *27+31*, *28*, *29*, *32*, *36*, *39*, *42*, *43*, *45*, *47*	*Lr1*, *2a*, *2b*, *2c*, *3*, *11*, *12*, *14a*, *14b*, *16*, *17b*, *18*, *20*, *21*, *22a*, *22b*, *23*, *26*, *30*, *33*, *35*, *37*, *38*, *40*, *44*, *48* and *49*

### Stability analysis

Stability analysis of 49 promising lines with two and three APR gene combinations were performed for grain yield under four different environments viz., Pantnagar, Varanasi, Powarkheda and Pune, using additive main effects and multiplicative interaction (AMMI) model. Proper data from other locations were not available and therefore not included in the AMMI analysis. AMMI analysis combines ANOVA with principal component analysis (PCA) to study genotype x environment interactions. AMMI biplots were prepared using PROC IML procedure of SAS version 9.3 as described by Zobel et al. [23] with mean and first two PC scores.

### Molecular maker screening for APR genes

#### DNA isolation

One hundred and ninety promising wheat accessions, selected based on field screening, were subjected to molecular characterization. Leaf samples were collected from 25 day-old seedlings for DNA extraction. Leaf samples were frozen immediately in liquid nitrogen and stored under deep freeze (-80°C) condition for the purpose of genomic DNA isolation. Genomic DNA was isolated using the cetyl-trimethyl ammonium bromide (CTAB) method described by Doyle and Doyle [24], with slight modifications.

#### DNA marker analyses

DNA markers were selected from the reference International Triticeae Mapping Initiative (ITMI) map [25]. The primers were dissolved in TE buffer according to the concentration of supplied primers, to create the working solution of 10μM concentration. Sequences and other information of the primers for markers associated with various leaf rust resistance genes are given in Table 2.

**Table 2:** Details of DNA markers used for screening the known APR genes in the selected wheat accessions.

Sl. No.	Gene	Marker	Sequence	Amplicon (bp)	Reference
1	*Lr34*+	*CsLv34*	GTT GGT TAA GAC TGG TGA TGG TGC TTG CTA TTG CTG AAT AGT	150	[16]
2	*Lr46*+	*Xwmc44*	AGG GAA AAG ACA TCT TTT TTT TC CGA CCG ACT TCG GGT TC	242	[39]
3	*Lr67*+	*Cfd71*	CAA TAA GTA GGC CGG GAC AA TGT GCC AGT TGA GTT TGC TC	214	[31]
4	*Lr68*	csGs	AAG ATT GTT CAC AGA TCC ATG TCA GAG TAT TCC GGC TCA AAA AGG	385	[33]

PCR reactions for the DNA markers were undertaken as described by Somers et al. [26], the purpose being to screen the accessions for the presence of APR genes. DNA amplification was carried out in a 96 well thermocycler (Eppendorf Thermal Cycler, Germany) in a volume of 20 μl., each containing 10 ng of genomic DNA, 0.5 μM of each primer, 0.2 mM of each dNTPs, 1.5 mM MgCl_2_, 10x PCR buffer and 1 U of Taq DNA Polymerase. The following PCR profile was followed: initial denaturation at 94°C for 3 min, followed by 45 cycles of 94°C for 1 min, 55 to 60°C (depending on primer) for 1 min, 72°C for 2 min with a final extension step of 7 min at 72°C and the samples were held at 4°C until samples were removed from the PCR for electrophoresis. The amplified PCR product was separated on 3% metaphor agarose gel at a constant voltage of 80 V for 2 to 3 hrs. The gels were stained with ethidium bromide solution, visualized and analyzed under the gel documentation system.

## Results

### Field screening for LTN and leaf rust reaction

Initially, 6,319 germplasm lines were screened for LTN and leaf rust reaction during the first season/year. Based upon the initial screening, 190 promising lines were selected which were further confirmed in the second season/year for the presence of LTN and leaf rust resistance under ten different locations, which falls under different wheat cultivating zones of India. Passport data of these 190 lines is given in S1 Table. LTN was scored on the flag leaf at post-flowering stage and leaf rust severity at Zadoks growth stage 87. Selected accessions displayed LTN of all categories ranging from low to very high. Each accession expressed varying degrees of LTN depending on the location. Accessions planted in locations which have relatively cool weather, such as Pantnagar, Ludhiana, Karnal, Varanasi, Kumarganj, and Wellington, displayed higher intensity/degree of LTN compared to warm weather locations, i.e. Vijapur, Powarkheda, Pune and Dharwad.

**Fig 1:**
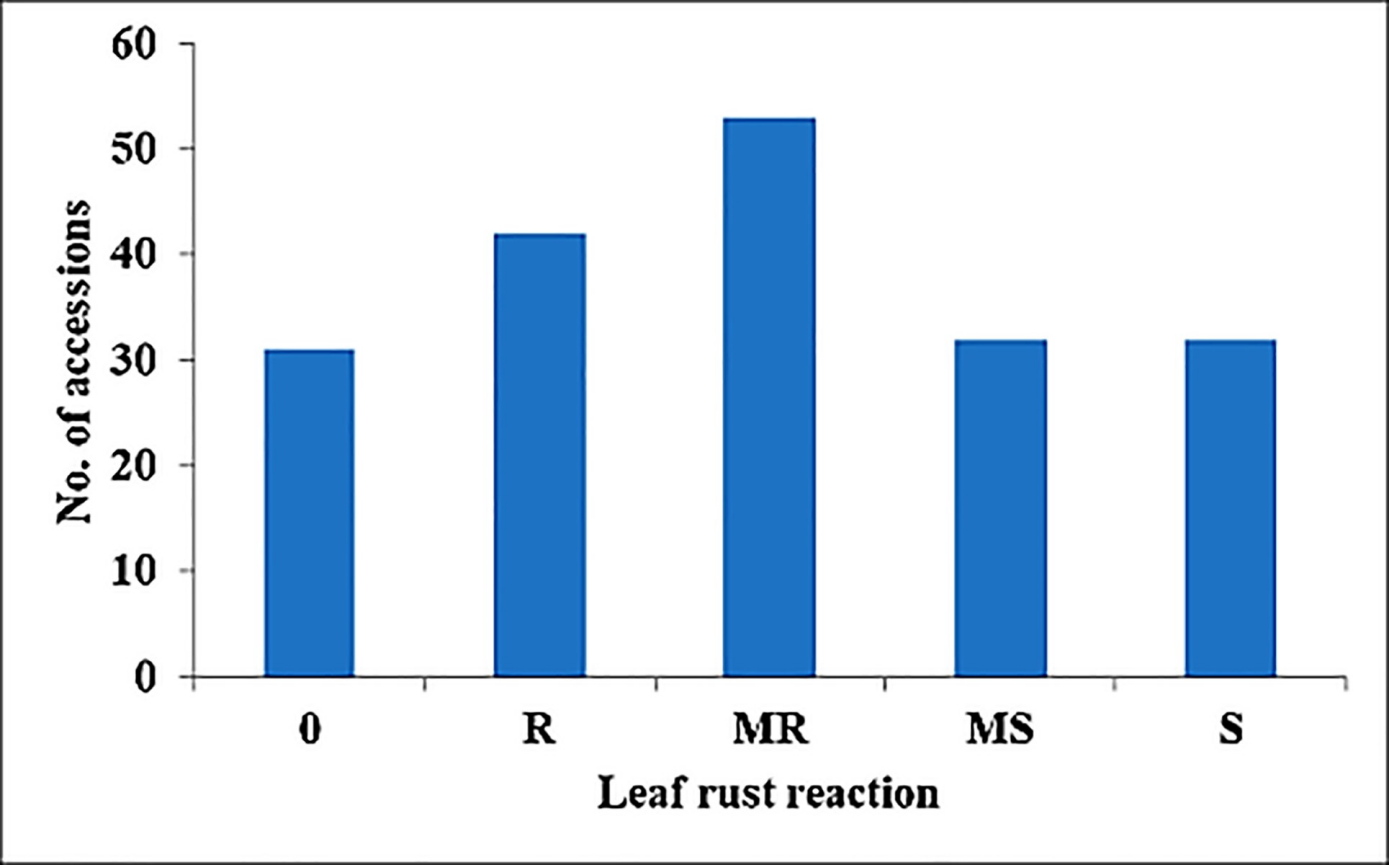
Leaf rust reaction* of the selected 190 wheat accessions. *R-Resistant; MR-Moderately resistant; MS-Moderately susceptible; S-Susceptible.

All kinds of leaf rust reaction types/pustules, i.e. Immune, R, MR, MS and S were observed in selected accessions. Of the 190 accessions, 31, 42, 53, 32 and 32 accessions showed immune, R, MR, MS and S reaction, respectively (Fig 1). Highest score and ACI of all 190 accessions are documented in Table 3.

**Table 3:** Highest score (HS) and average coefficient of infection (ACI) based on leaf rust reaction of 190 germplasm accessions at 10 locations.

Sl. No.	Accession No.	Highest Score (HS)	Average Coefficient of Infection (ACI)	Sl. No.	Accession No.	Highest Score (HS)	Average Coefficient of Infection (ACI)	Sl. No.	Accession No.	Highest Score (HS)	Average Coefficient of Infection (ACI)
1	IC11659	0	0.0	66	IC145884	20MS	9.4	131	IC290162	10R	3.4
2	IC11670	20R	8.3	67	IC145916	20MS	6.3	132	IC290168	20MS	8.4
3	IC128565	0	0.0	68	IC145951	10R	2	133	IC290173	10MR	8.2
4	IC25270	20R	6.7	69	IC145977	10R	1.4	134	IC290175	20S	8.8
5	IC73591	0	0.0	70	IC252370	20S	8.8	135	IC290176	10MS	8.8
6	IC73593	0	0.0	71	IC252392	20MS	8.1	136	IC290177	20MR	1.6
7	IC75313	0	0.0	72	IC252431	20MR	2.7	137	IC290178	10R	2.8
8	IC75314	10S	6.3	73	IC252432	20R	15.0	138	IC290182	20S	8.4
9	IC82194	20MR	5.3	74	IC252433	10MR	7.7	139	IC290184	10R	3.6
10	IC111667	20MS	6.7	75	IC252439	10R	4.2	140	IC290186	20MR	4.8
11	IC111668	0	0.0	76	IC252441	20MR	6.7	141	IC290190	5R	1.0
12	IC111686	10R	3.3	77	IC252443	20MS	6.0	142	IC290197	20MS	5.6
13	IC111687	0	0.0	78	IC252444	20R	8.0	143	IC290208	20MR	8.4
14	IC111688	0	0.0	79	IC252445	20S	7.5	144	IC290215	10S	8.8
15	IC111691	0	0.0	80	IC252448	20R	8.3	145	IC290217	20MS	7.2
16	IC111692	0	0.0	81	IC252450	20MR	8.3	146	IC290222	20MR	4.0
17	IC111693	5R	1.7	82	IC252453	0	0.0	147	IC290226	20MS	8.8
18	IC111694	20S	6.7	83	IC252455	20MR	5.0	148	IC290227	10MR	0.8
19	IC111701	10MR	3.3	84	IC252456	10MR	2.5	149	IC290231	10MS	4.8
20	IC111731	20R	6.7	85	IC252457	20S	10	150	IC290241	10MR	0.8
21	IC111771	0	0.0	86	IC252458	0	0.0	151	IC290242	20R	4.4
22	IC111783	20S	6.7	87	IC252459	5MR	0.8	152	IC290243	20MR	4.8
23	IC111787	10R	3.3	88	IC252469	5R	0.8	153	IC290244	10MR	0.8
24	IC111888	0	0.0	89	IC252472	0	0.0	154	IC290258	5MR	0.4
25	IC111892	0	0.0	90	IC252490	0	0.0	155	IC290261	5MR	0.4
26	IC111905	20S	6.7	91	IC252497	0	0.0	156	IC290262	20S	8.1
27	IC111912	20MS	10.0	92	IC252499	5R	0.9	157	IC290264	20R	5.6
28	IC111918	20S	8.0	93	IC252520	10MR	1.7	158	IC290280	0	0.0
29	IC111919	20MS	6.7	94	IC252541	20MS	5.8	159	IC290281	0	0.0
30	IC128179	20S	10.0	95	IC252542	10MR	1.7	160	IC290298	10MR	2.8
31	IC128457	10MR	6.0	96	IC252547	10MR	1.7	161	IC290299	5MR	0.8
32	IC128507	20S	6.7	97	IC252591	0	0.0	162	IC290302	10MR	0.8
33	IC128520	20S	7.6	98	IC252611	0	0.0	163	IC290305	20S	5.6
34	IC128521	10MR	4.0	99	IC252629	5R	0.8	164	IC290309	0	0.0
35	IC128524	10R	8.4	100	IC252650	0	0.0	165	IC290311	10MR	2.8
36	IC128525	20S	8.6	101	IC252673	0	0.0	166	IC290314	10R	2.2
37	IC128526	10R	7.1	102	IC252676	10R	2.2	167	IC290316	10R	2.0
38	IC128553	10R	4.5	103	IC252684	10S	4.7	168	IC290325	10R	9.6
39	IC128555	20S	8.5	104	IC252686	5R	3.3	169	IC290326	10MR	4.4
40	IC128587	20MS	6.5	105	IC252706	20MR	7.0	170	IC290327	20R	4.2
41	IC128590	20S	6.5	106	IC252723	20S	6.7	171	IC290329	10S	8.8
42	IC128592	20MR	6.5	107	IC252725	20MR	6.9	172	IC290342	20MS	9.6
43	IC128594	20MS	6.5	108	IC252767	10MR	0.7	173	IC290065	20MR	4.0
44	IC128619	20MR	6.5	109	IC252768	0	0.0	174	IC310106	20MR	4.4
45	IC128624	20S	7.1	110	IC252818	10R	2.2	175	IC310120	20MS	4.8
46	IC128629	20R	7.1	111	IC252819	20R	6.8	176	IC310124	20S	8.2
47	IC128631	10MR	6.0	112	IC252892	0	0.0	177	IC316100	10MR	2.4
48	IC128637	20MS	7.7	113	IC252983	10MR	1.8	178	IC321153	20R	4.4
49	IC128638	0	0.0	114	IC260877	5R	1.0	179	IC335670	5MR	0.4
50	IC128650	20S	7.9	115	IC279320	10MR	5.0	180	IC335671	10MR	0.8
51	IC128652	10MR	5.3	116	IC279321	20S	8.3	181	IC335683	10MR	0.8
52	IC128654	20S	8.0	117	IC279333	10R	2.7	182	IC335704	10MS	2.0
53	IC128656	20MS	9.8	118	IC279875	0	0.0	183	IC416080	5MR	0.4
54	IC128692	20MS	4.9	119	IC281566	0	0.0	184	IC416082	20MR	4.4
55	IC138364	20S	7.8	120	IC290022	10R	1.7	185	IC416083	10MS	1.6
56	IC138426	10MS	5.4	121	IC290025	10MR	1.7	186	IC416084	10MR	2
57	IC138479	0	0.0	122	IC290039	10MR	0.8	187	IC416092	10R	1.4
58	IC138521	20MS	15.7	123	IC290046	20MS	7.7	188	IC416094	20MS	8.8
59	IC138524	20R	10.3	124	IC290057	10MR	7.0	189	IC416281	20MS	8.1
60	IC145331	20S	15.3	125	IC290058	20S	10.0	190	IC427210	20R	2.7
61	IC145602	10MR	8.8	126	IC290087	20MS	8.3	191	Avocet+*Lr34*	30MS	24.6
62	IC145780	20MR	14.5	127	IC290098	20MS	8.3	192	Avocet+*Lr46*	40S	29.0
63	IC145808	10S	10.3	128	IC290150	5R	0.7	193	Avocet+*Lr67*	40S	31.7
64	IC145811	10MS	9.5	129	IC290154	10R	1.8				
65	IC145882	10MS	5	130	IC290157	20S	6.7				

**Fig 2:**
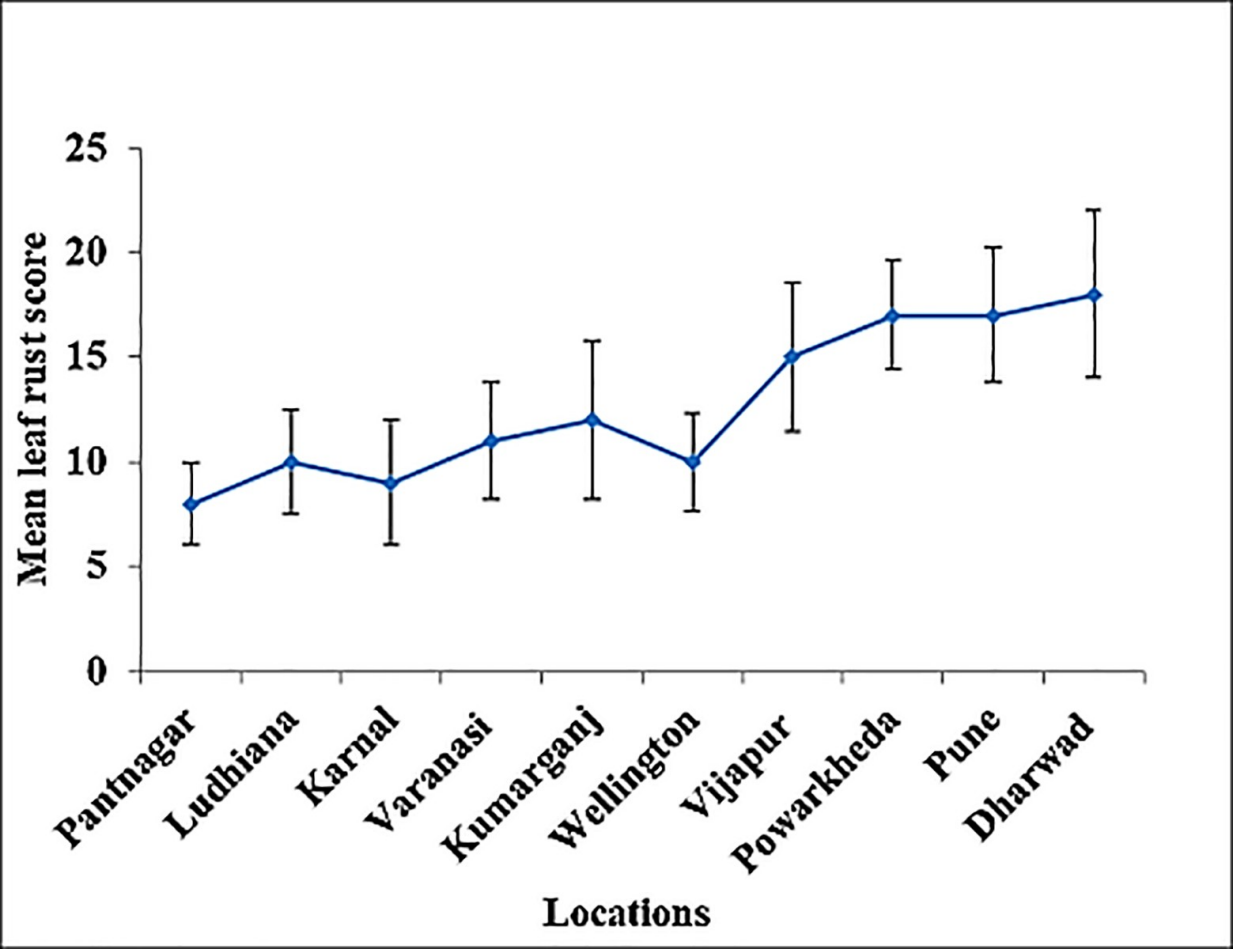
Mean leaf rust infection of wheat accessions grown at 10 different locations across the climatic zones of India.

Analyses of location-wise data revealed that accessions grown in comparatively cooler locations (Pantnagar, Ludhiana, Karnal, Varanasi, Kumarganj and Wellington) had low leaf rust infection compared to warmer locations (Vijapur, Powarkheda, Pune and Dharwad) (Fig 2). Based on all 10 locations, out of 190 accessions, 153 showed ACI less than ≤10.00, 6 showed ACI >10.0, while thirty- one accessions were completely free of leaf rust and displayed immune reaction. Only accessions with a high level of APR (0–20) were taken into account. Since the leaf rust severity was less in cooler locations, it was reflected as low ACI than the warmer ones. In most of the resistant accessions leaf rust severity progressed at a retarded rate, resulting in intermediate to low disease levels (slow rusting) except the immune accessions. In contrast, Avocet+*Lr34*, Avocet+*Lr46* and Avocet+*Lr67* displayed susceptible reaction under disease pressure. Accessions, which displayed the presence of LTN and resistant reaction for leaf rust in all the locations were taken into consideration for further molecular marker screening.

### Molecular marker screening for APR genes

Accessions selected based on LTN and leaf rust reaction were further subjected to molecular marker confirmation for the presence of known APR genes viz., *Lr34*+, *Lr46*+, *Lr67*+ and *Lr68*. Each marker reaction was repeated twice to ensure the reproducibility of the amplification pattern, without alteration of protocol. Accessions screened for markers linked to leaf rust resistance genes indicated the presence of gene(s) either alone or in combination or none. Out of 190 accessions, 51, 18, 13 and 7 accessions possessed the genes *Lr34*+, *Lr46*+, *Lr67*+ and *Lr68* alone, respectively, while 20, 3, 8, 6, 2 and 1 accessions had the genes in combination as *Lr34*+ *Lr46*, *Lr34*+ *Lr67*, *Lr34*+ *Lr68*, *Lr46*+ *Lr67*, *Lr46*+ *Lr68* and *Lr67*+ *Lr68*, respectively (S2 Table). However, nine accessions consisted of three genes in combination, for example: four accessions had *Lr34*+*Lr46*+*Lr67*; three had *Lr34*+*Lr46*+*Lr68*; and one each had *Lr34*+*Lr67*+*Lr68* and *Lr46*+*Lr67*+ *Lr68*. None of them carried all the four genes together and fifty-two accessions carried none of these APR genes.

**Fig 3:**
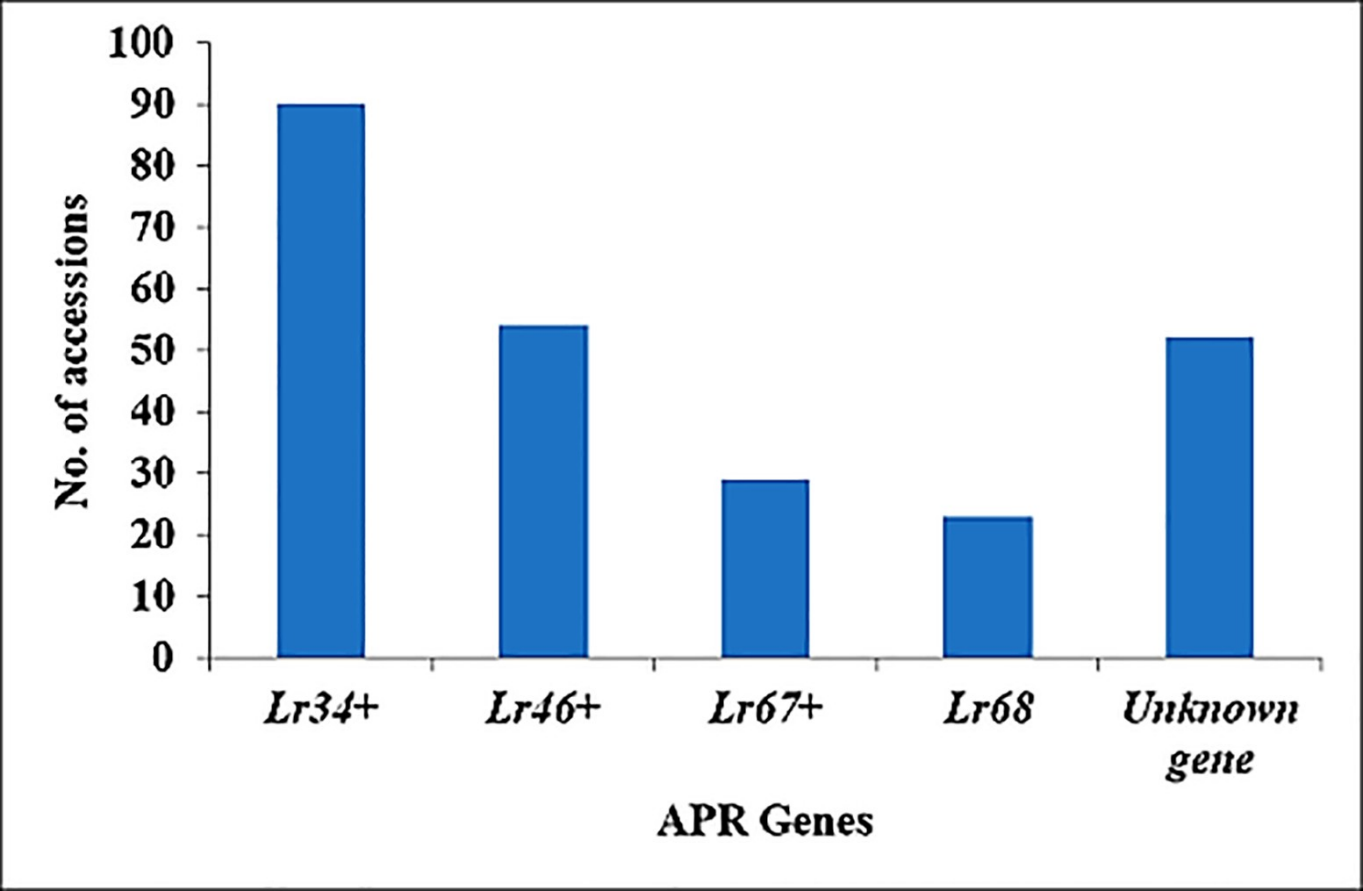
Distribution of APR genes among the selected wheat accessions.

**Fig 4:**
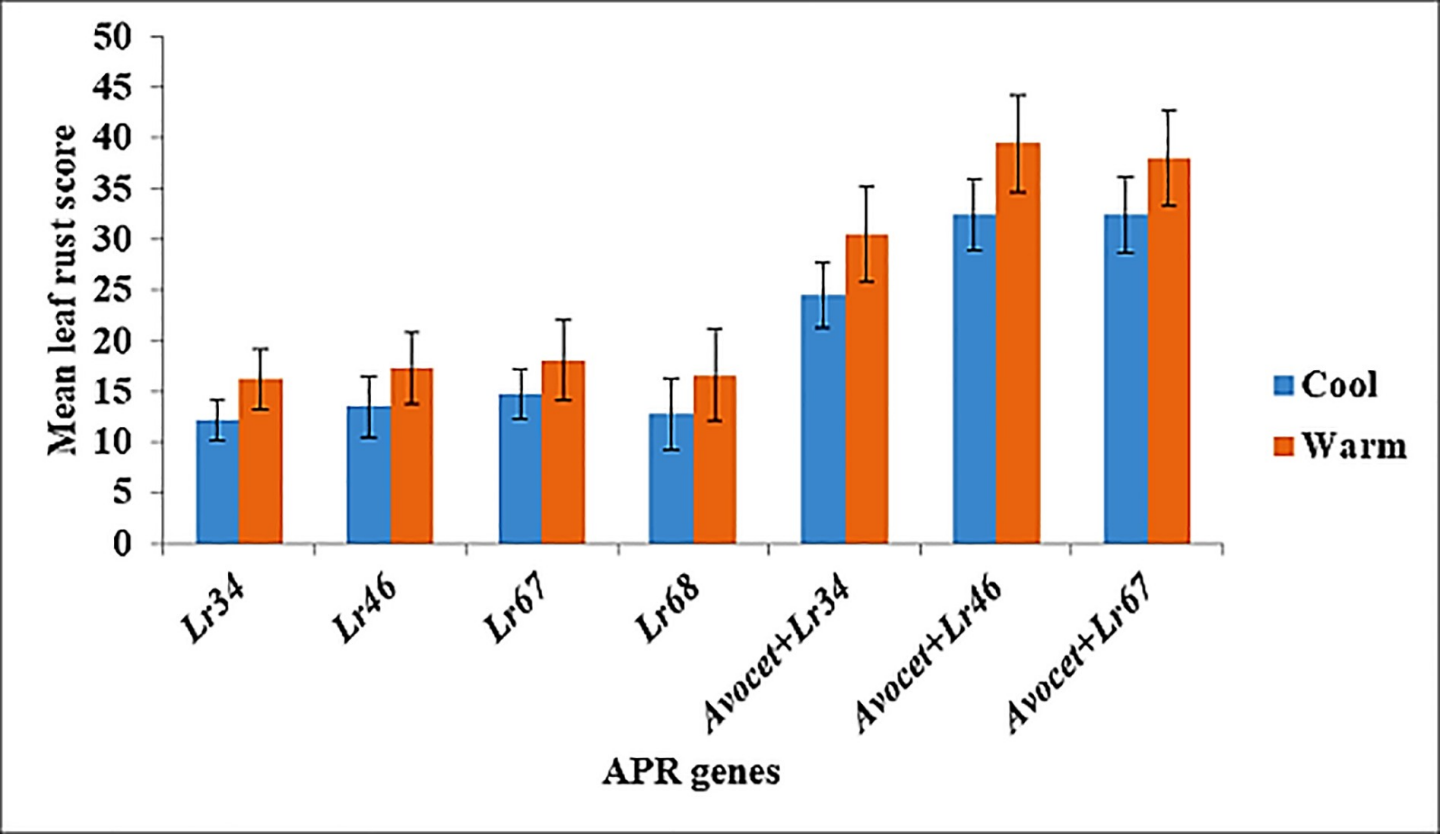
Effect of temperature on the effectiveness of different APR genes against leaf rust severity. Error bar displays the standard error of the mean.

Total number of accessions with APR genes alone and in combination with other APR genes included 90, 54, 29 and 23 for *Lr34*+, *Lr46*+, *Lr67*+ and *Lr68*, respectively (S2 Table; Fig 3). Effect of individual APR gene on leaf rust resistance revealed that environmental factors, particularly temperature, play a vital role in resistance. Resistance due to APR gene in accessions grown in cooler locations had 24%, 22%, 17% and 23% less leaf rust severity than accessions grown in warmer locations for *Lr34*+, *Lr46*+, *Lr67*+ and *Lr68*, respectively (Fig 4). Similarly, in isogenic control, 20%, 17% and 15% reduction in leaf rust severity was observed for Avocet+*Lr34*, Avocet+*Lr46* and Avocet+*Lr67*, respectively (Fig 4). Representative molecular profiling of 48 wheat genotypes with linked DNA markers (a) *CsLv34 (Lr34) and (b) Cfd71(Lr67)* used for screening the known APR genes is given in S1 Fig.

**Fig 5:**
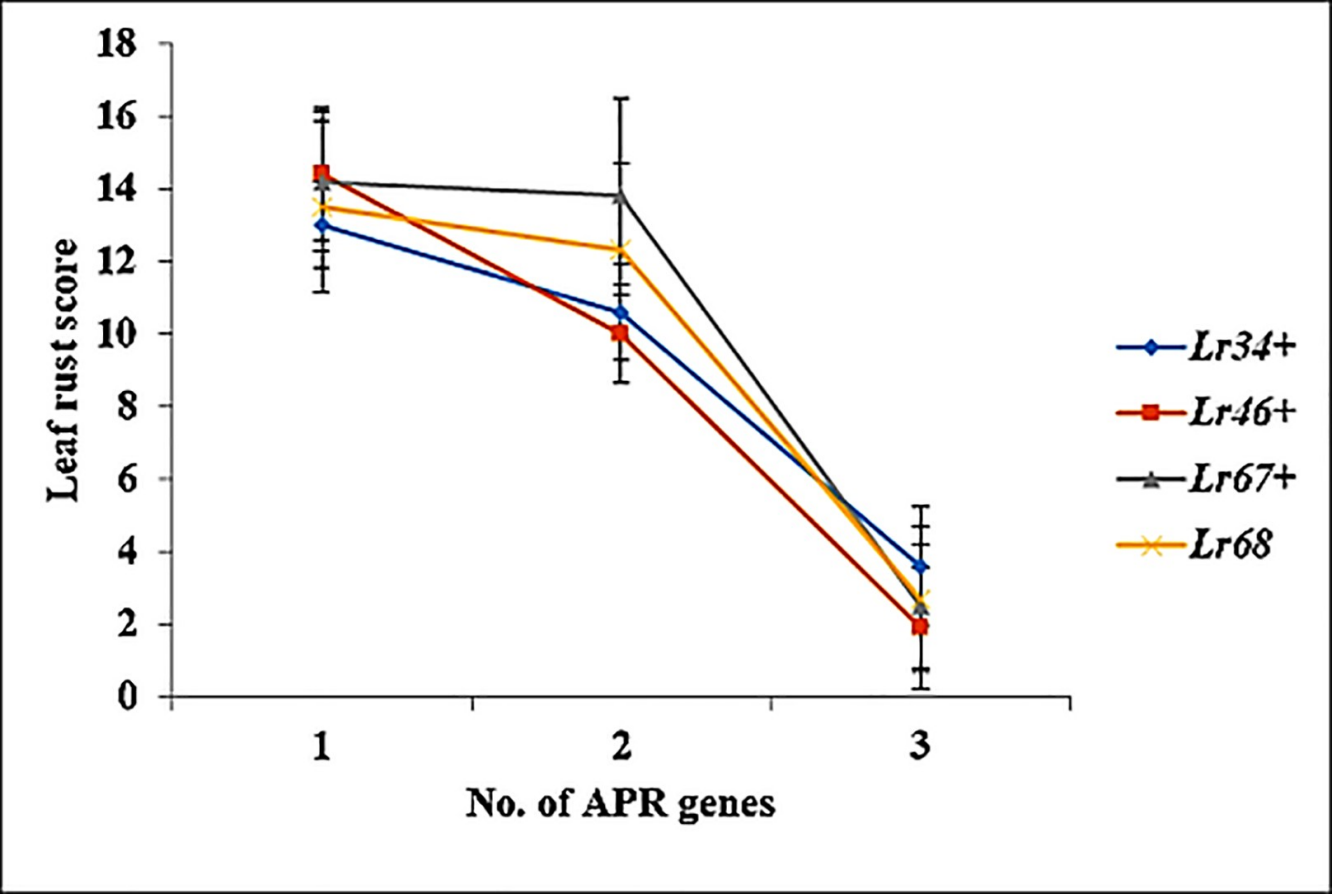
Leaf rust reaction with respect to number of APR genes in wheat accessions. Error bar displays the standard error of the mean.

Accumulation of APR genes in accessions improved the level of resistance due to additive gene action. For example, in the case of *Lr34*+, the presence of *Lr34+* alone gave a mean rust score of 13. The addition of one more APR gene (either *Lr46+* or *Lr67+*) reduced the mean rust score to 10, while adding two more APR genes made the accession highly resistant or nearly immune (Fig 5). Background effect of other unknown rust resistance genes present in these accessions also played a vital role in providing resistance since Avocet+*Lr34* (isogenic control) indicated susceptible reaction. Similarly, the remaining APR genes evidenced higher resistance in combination with other APR genes (Fig 5).

### Adaptability

**Fig 6:**
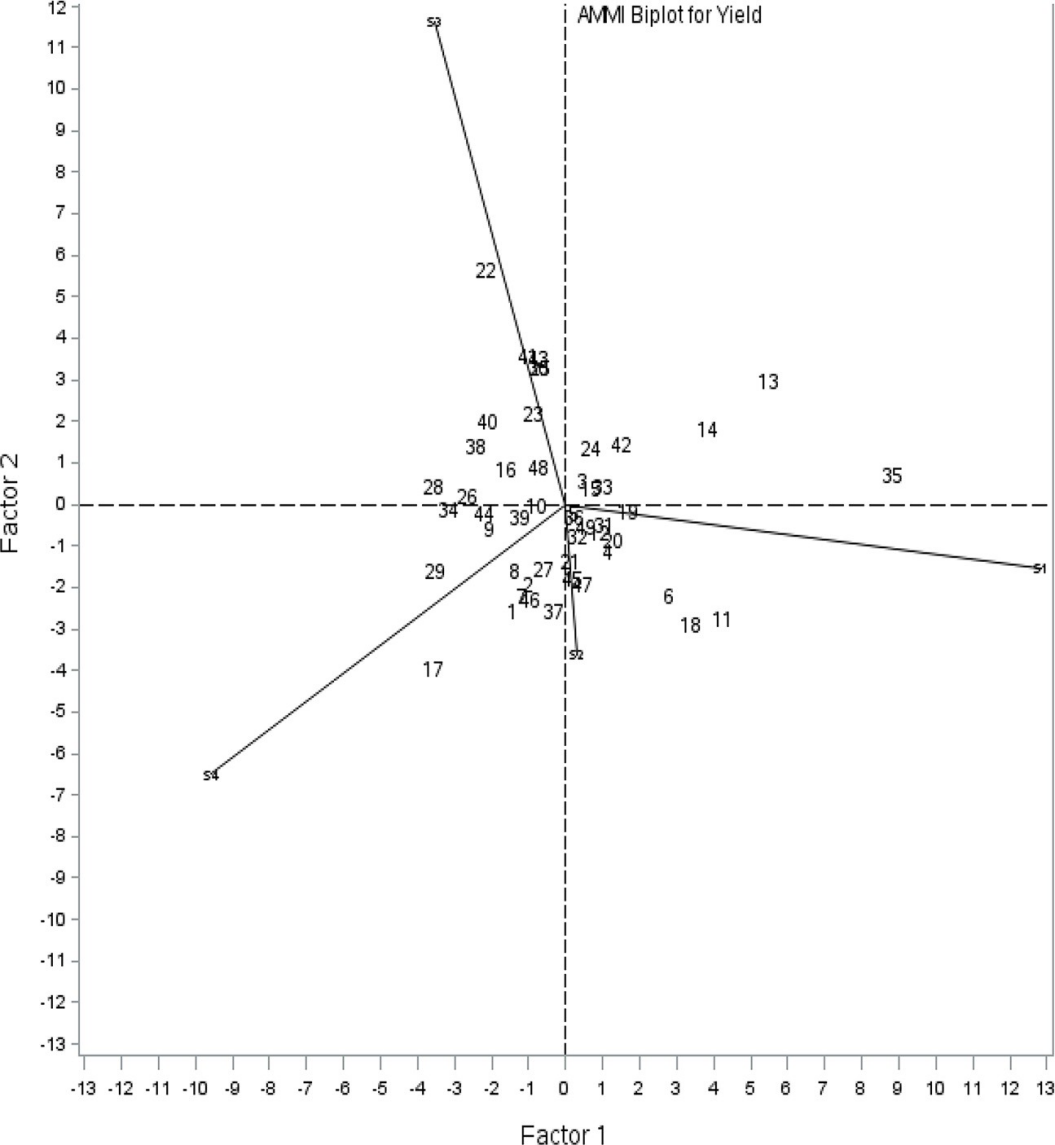
AMMI biplot showing stability of promising genotypes across four environments for grain yield.

The identified 49 accessions with a combination of 2 and 3 leaf rust resistant genes -*Lr34+*, *Lr46+*, *Lr67+* and *Lr68* with low disease severity across all the environments were further subjected to stability analysis. To analyze whether these germplasm accessions perform in a stable way for grain yield in the screening environments, additive mean effects and multiplicative interaction (AMMI) analysis was carried out. AMMI analysis was conducted employing Gollob’s test in which the first two principal components (PC) explained 61.91% and 38.08% variation, respectively. The graphical method was employed utilizing two PC to investigate environmental variation and interpret the G × E interaction for grain yield (Fig 6). The wheat germplasm closer to the origin of biplot was stable across the four test locations. Accessions IC073591, IC128638, IC11659, IC252392, IC252459, IC290039, IC290314 and IC128650 had low PCA scores and clustered near the biplot origin, indicating wide adaptability for grain yield.

The lines connecting the biplot origin and the margins for the environment are called environment vectors. The angle between two environment vectors relates to the correlation coefficient between them. Based on the angles of the environment vectors, locations Powarkheda (S2) and Varanasi (S4) have relatively good correlation between them, which meant that they are mostly similar in performance. The observation related to length of the environment vector depicts standard deviation within each environment. A critical assessment of biplot revealed that location Pune (S3) has large genotype X environmental interaction and greater discriminating power for grain yield followed by Pantnagar (S1), Varanasi (S4), and Powarkheda (S2). Germplasm accession viz., IC279875 was specifically adapted to Pantnagar (S1), IC290022 to Pune (S3); IC252445 to Varanasi (S4); IC252983, IC290168, IC252450, and IC290309 for Powarkheda (S2), regarding grain yield (Fig 6).

## Discussion

The present study was conducted to identify the Indian wheat germplasm accessions carrying APR genes. Accessions were screened at ten different locations, i.e. Pantnagar (Uttarakhand), Ludhiana (Punjab), Karnal (Haryana), Varanasi (Uttar Pradesh), Kumarganj (Uttar Pradesh), Vijapur (Gujarat), Powarkheda (Madhya Pradesh), Pune (Maharashtra), Dharwad (Karnataka), and Wellington (Tamilnadu). Initially, to select from a large number of accessions, they were evaluated for LTN, a phenotypic trait linked to adult plant leaf rust resistance genes viz., *Lr34*+, *Lr46*+, *Lr67*+ and *Lr68*. LTN is characterized by presence of necrosis on flag leaf tips which extend to a few centimetres along the leaf edges [27, 14]. Accessions expressed LTN in varying degrees from low to very high. Leaf tip necrosis is considered an innate defense mechanism and is known to occur spontaneously in wheat lines having *Lr34* gene [28, 15]. While the genetic basis of this mechanism is not clearly understood, it is suggested that *Lr34* might some way alter the physiology of flag leaf, thus making it a less desirable for the pathogen to grow and establish [29]. Although *Lr34/Ltn1* was the first gene of wheat associated with LTN, some other genes including the genes *Lr46/Ltn2* [30], *Lr67/Ltn3* [31, 32] and *Lr68* [33] were also found to confer LTN in varying degrees.

In our study, stable expression of LTN was not noticed; the same accessions expressed different levels of LTN at different locations, thereby confirming the quantitative expression. Low temperature favors the expression of LTN, which is pronounced in accessions grown in cooler locations. Similarly, stronger LTN was observed in seedlings of *Lr34* transgenic plants exposed to cold temperature, than untreated plants, which implies that the increase in LTN is due to elevated *Lr34* expression levels at low temperature [34]. Differential expression of LTN associated with APR genes, *Lr34*, *Lr46*, *Lr67* and *Lr68* is a result of interaction with the environment [18]. Expression of LTN is not an attractive trait, still its role as a phenotypic marker for APR is valuable, i.e. used as a preliminary screening method to detect the presence of APR while screening large germplasm accessions. It may also have a slight effect in reducing grain yields in some environments [35]. Inspite of this fact, many high yielding wheat cultivars possess this trait [13].

Simultaneously, these accessions were also screened for adult plant response to leaf rust under disease pressure; infection response was observed from immune to R/MR/MS/S. In India, wheat growing areas are divided into six zones while the ten locations of our study are located in five wheat cultivation zones except the northern hill zone (minor zone). These five zones contribute upto 95% of wheat production of India and leaf rust is a common problem in all these zones. Ludhiana, Karnal and Pantnagar occupy the North West Plain Zone (NWPZ), which is considered to be the “wheat basket of India” where wheat is grown under irrigated conditions with adequate moisture supply and cool weather. A more or less similar situation prevails in North East Plain Zone (Varanasi and Kumarganj) with humid weather. Leaf rust pathotypes 77–5 (121R 63–1), 77-9(121R60-1) and 104–2 (21R55) are the dominant pathotypes of these zones. In the case of Central zone (Vijapur and Powarkheda) and Peninsular zone (Pune and Dharwad) which are either rain-fed or restricted irrigation conditions with dry weather, in addition to pathotypes 77–5 (121R63-1), 77-9(121R60-1) and 104–2 (21R55); pathotypes like 77–1 (109R 63), 104-1(21R31-1) and 104-3(21R63) are prevalent. Wellington is the hot spot for leaf and stem rust wherein cool weather prevails throughout the year and the major leaf rust pathotypes are 77–5 (121R63-1) and 77-9(121R60-1). These accessions were resistant to major pathotypes (77–5 & 77–9) and other minor pathotypes (77–1, 104–1, 104–2 & 104–3) which could add to the diversity of resistance already being deployed/present in these zones. Comparatively, leaf rust severity is less in cooler locations than warmer ones, which confirms the role of environment in the expression of APR genes/quantitative nature of APR genes [36].

Level of APR was determined on the basis of average coefficient of infection (ACI) values. ACI of the majority of accessions were low (≤10) which implies the ability of an accession to provide non-specific resistance to the pathotypes across all the tested locations. Accessions which were resistant to leaf rust, along with LTN in all the locations were selected, which enabled the filtering of accessions from 6,319 to 190. Since, these accessions were screened in diverse climatic conditions ranging from cool to warm weather and from irrigated to rain-fed conditions, these accessions could be a potential parent/donor for breeding leaf rust resistant varieties in these zones.

One hundred and ninety accessions selected based on LTN and leaf rust reactions were furtherconfirmed for the presence of known APR genes using molecular/DNA markers. Of the four APR genes screened using the DNA marker, *Lr34*+ was the most common gene present in 47% (90 accessions) of the accessions as single and in combination with other APR genes. *Lr34*+ is the most studied, possibly the most effective and cloned slow rusting leaf rust resistance gene located on chromosome 7DS. It has maintained its moderate effectiveness for over 60 years of use [37]. *Lr34*+ was first characterized in South American cultivar, Frontana [14] and present in landraces of China including Chinese spring. Dakouri et al. [38] suggested that *Lr34*+ most likely originated from Asia, specifically China or Japan. The presence of *Lr34*+ in Indian wheat germplasm is mainly attributed to the selections made from CIMMYT international nurseries or the use of CIMMYT germplasm as one of the parents in the hybridization program or else through a Chinese-origin germplasm. Reduced pustule size observed in *Lr34*+ accessions may be one of the factors imparting slow rusting phenomenon. *Lr34*+ in combination with other APR genes is more effective and could provide immune reaction in accessions like IC128565, IC73591, IC11171, etc.

*Lr46*+ was present in 28% (54 lines) accessions that were evaluated and is located on chromosome 1BL [10, 39]. It was first identified in the CIMMYT derived Mexican variety, Pavon 76. In comparison, *Lr46*+ was less effective than *Lr34*+. The presence of *Lr46*+ in Indian wheat germplasm is mainly due to the popular wheat cultivar, PBW343, which has been extensively used in the wheat-breeding program. Lagudah [36] reported that warmer temperature associated with very late field sowing were considered the likely cause of ineffectiveness of *Lr34*+ and *Lr46*+. The same could be observed in accessions in the Central and Peninsular zones which have warmer weather; higher leaf rust score was observed in these locations compared to North West and North East Plain Zone and Wellington which are relatively cooler.

Fifteen per cent (29 accessions) of the accessions carried *Lr67*+, which is located on chromosome 4DL [32, 31]. Earlier studies revealed that *Lr67*+ supposedly originated from India [40, 41]. However, in the present study few lines were detected with *Lr67*+. This may be due to *Lr67*+ accessions having comparatively lower LTN expression than *Lr34*+ and *Lr46*+,thereby resulting in higher probability of ignoring the accessions during selection for LTN. Careful examination of LTN is required for selection of *Lr67*+ lines, which may be confused with leaf tip drying. Moreover, expression of *Lr67*+ is variable depending on the background [42].

A relatively new addition to the APR gene category is *Lr68*, which is present in 12% (23 accessions) of accessions. *Lr68* is located in chromosome 7BL and characterized initially in CIMMYT wheat, Parula [33]. Expression of *Lr68* depends on temperature wherein lower temperature favors the expression [33]. Since some of the locations had warm weather, few lines could be identified with *Lr68*. It was observed that individually, APR genes do not confer adequate resistance under disease pressure. However, a combination of two APR genes improved the resistance level, while a combination of three APR genes provided a high level of resistance (Fig 5) as reported by Singh et al. [43].

Fifty-two accessions were not identified with any of the APR genes evaluated. These accessions were deemed to carry potentially new sources of APR. Of these, nine accessions showed immune reaction while the remaining lines displayed varying levels of resistance. Variability in leaf rust reaction is likely due to differences in environment (temperature) and growth condition (irrigated/rain-fed) across the locations. APR genes provide a low level of resistance in field conditions under high disease pressure, so it is possible that lines showing low resistance could carry novel APR genes. In addition to the APR genes screened in this study, a number of other race-specific APR have been catalogued including *Lr12*, *Lr13*, *Lr22a*, *Lr22b*, *Lr35*, *Lr37*, *Lr48* & *Lr49* [11, 44, 9] which need to be confirmed in these lines, some novel APR genes might be identified in these lines. Moreover, immune accessions indicate the presence of ASR genes or a combination of ASR and APR genes in the background. Therefore, to determine whether the resistance is due to novel genes, seedling reaction against prevalent pathotypes followed by mapping is required.

The AMMI model is a widely used statistical tool in the analysis of multi-environmental trials. It can be used to understand and structure interactions between genotypes and environments. The AMMI analysis can be very useful in understanding the complex genotype x environment interaction, which includes delineating mega environments or identification of productive cultivars with wide adaptability, as well as delimit the agronomic zoning of cultivars with specific adaptability [45, 46]. Accessions with three gene combinations, viz., IC073591 (*Lr34*+*Lr46*+*Lr67*) and IC128638 (*Lr46*+*Lr67*+*Lr68*) and two gene combinations, IC11659 (*Lr34*+*Lr46*), IC252392 (*Lr34*+*Lr46*), IC252459 (*Lr34*+*Lr67*), IC290039 (*Lr34*+*Lr68*), IC290314 (*Lr46*+*Lr68*) and IC128650 (*Lr67*+*Lr68*) displayed wider adaptability and stability for yield and yield attributing traits across locations. Therefore, they can be used as parents for the introgression of APR genes without compromise on yield or with minimal linkage drag. However, a few accessions performed better in specific locations like IC279875 (*Lr34*+*Lr68*) to Pantnagar, IC290022 (*Lr34*+*Lr46*) to Pune, IC252445 (*Lr34*+*Lr68*) to Varanasi and IC252983 (*Lr34*+*Lr68*), IC290168 (*Lr34*+*Lr68*), IC252450 (*Lr46*+*Lr67*), IC290309 (*Lr46*+*Lr68*) to Powarkheda. These accessions are adapted to specific areas which can be used for the improvement of cultivars of particular locations.

Accessions displayed diverse disease reaction, i.e. immune, resistant, susceptible and intermediate types, irrespective of the presence of single gene or combinations of genes. Cooler temperature/locations favor the expression of APR genes, which is reflected in low leaf rust severity and ACI than warmer temperature/locations. This is due to the fact that APR gene expression is sensitive to environmental conditions, because of: firstly, the quantitative nature of the resistance; and secondly, the genetic background of the germplasm. Since the environment/weather conditions of the screening location showed variation, germplasm lines also displayed diverse disease reactions. In recent years, rust resistance in the wheat improvement program is directed towards durable resistance based on adult plant resistance (APR)/minor genes to avoid the frequent breakdown of major gene-based resistance by new emerging pathotypes. The purpose of durable resistance based on minor genes is to remain effective in a cultivar during its widespread cultivation for a long sequence of generations or period of time, in an environment favorable to a disease or pest [47].

These accessions were collected from previously mentioned wheat cultivated regions of India wherein leaf rust is a common problem. These collections include land races, old cultivars, obsolete varieties, cultivars, etc. For thousands of years, wheat cultivation has been practiced in this region but it has also created the opportunity and conditions for the rust pathogen to co-evolve with wheat. Hitherto, the majority of cultivars in these regions were introgressed with major/race-specific rust resistant genes, i.e. *Lr13*, *Lr23*, *Lr24*, *Lr26*, etc., due to human intervention and no/little emphasis was given for APR genes. Moreover, presence of ASR/ major gene masks the effect/presence of APR gene in a genetic background. Thus, the directional selection in favour of the ASR/major rust resistance gene kept the minor genes at bay. So, when these germplasm lines were subjected to molecular marker analyses, presence of APR gene got detected. After achieving a certain level of resistance, nature of gene is more important rather than number of genes. APR genes, *Lr34*, *Lr46* and *Lr67* have been studied extensively and it had pleiotrophic association with stem and stripe rust resistance, i.e. *Lr34/Yr18/Sr57/Pm38/Ltn1*, *Lr46/Yr29/Sr58/Pm39/Ltn2* and *Lr67/Yr46/Sr55/Pm46/Ltn3* providing resistance not only to leaf rust, but also to stem and stripe rust. Presently, it is being extensively used in the wheat improvement program in India and worldwide. However, the eight germplasm accessions with three and two APR gene combinations having wider adaptability could be exploited for broadening the genetic base of the cultivated wheat to provide durable resistance. Closely linked molecular markers could be useful in the marker assisted selection or mobilization of these genes in elite wheat backgrounds.

Improvement of any crop lies in exploring and exploiting the rich gene pools available in its cultivated forms, land races, wild relatives and related genera. The conservation of a resource only becomes important if the resource has or acquires recognized value. This study highlights the importance of wheat germplasm in providing a durable solution for the evolving rust pathogen. Pyramiding of APR genes such as *Lr34*+, *Lr46*+, *Lr67*+ and *Lr68* is expected to confer near immunity, which presents an attractive option to breeders for durable multi-pathogen resistance to leaf, stem and stripe rust. Availability of robust and diagnostic molecular markers for the APR genes would speed up the process of durable rust resistance breeding. Accessions deemed to potentially carry new sources of APR genes require more detailed study.

## Conclusions

Rust pathogens have the ability to evolve new pathotypes rapidly with high reproductive rate and to spread quickly are a major threat to food security. Screening and deployment of genes warrants searching for additional genes, which confer race non-specific resistance to provide durable resistance. Germplasm collected from different agro-ecological regions at different points of time provide an opportunity to bio-prospect for such genes. The genetic base for leaf rust resistance among cultivars could be broadened and diversified through the use of resistant accessions identified in the study as donors in wheat improvement programmes.

## Supporting information

S1 Table Passport data of 190 leaf rust resistant Indian wheat accessions. (DOC)

S2 Table Adult plant resistance (APR) genes in the leaf rust resistant accessions. (DOC)

S1 Fig Representative molecular profiling of 48 wheat genotypes with linked DNA markers (a) *CsLv34 (Lr34) and (b) Cfd71(Lr67)* used for screening the known APR genes.
(DOC)
