# Supplementary material for: Mining of Indian wheat germplasm collection for adult plant resistance to leaf rust
Source: PLoS One. 2019 Mar 28;14(3):e0213468. doi: 10.1371/journal.pone.0213468 (PMC6438482; doi:10.1371/journal.pone.0213468)
Supplement: S1 Table — (DOC) [file pone.0213468.s001.doc]

| **S1 Table. Passport data of 190 leaf rust resistant Indian wheat accessions** | | | | |
| --- | --- | --- | --- | --- |
| **S. No.** | **IC Number** | **District** | **State** |  |
| 1 | IC11659 | -* | - |  |
| 2 | IC11670 | - | - |  |
| 3 | IC128560 | Kanpur | Uttar Pradesh |  |
| 4 | IC252700 | - |  |  |
| 5 | IC73591 | Bhowali, Nainital | Uttar Pradesh |  |
| 6 | IC73593 | Bhowali, Nainital | Uttar Pradesh |  |
| 7 | IC75313 | - | Uttar Pradesh |  |
| 8 | IC75314 | - | Uttar Pradesh |  |
| 9 | IC82194 | - | - |  |
| 10 | IC111667 | - | - |  |
| 11 | IC111668 | - | - |  |
| 12 | IC111686 | - | - |  |
| 13 | IC111687 | - | - |  |
| 14 | IC111688 | - | - |  |
| 15 | IC111691 | - | - |  |
| 16 | IC111692 | - | - |  |
| 17 | IC111693 | - | - |  |
| 18 | IC111694 | - | - |  |
| 19 | IC111701 | - | - |  |
| 20 | IC111731 | - | - |  |
| 21 | IC111771 | - | - |  |
| 22 | IC111783 | - | - |  |
| 23 | IC111787 | - | - |  |
| 24 | IC111888 | - | - |  |
| 25 | IC111892 | - | - |  |
| 26 | IC111905 | - | - |  |
| 27 | IC111912 | - | - |  |
| 28 | IC111918 | - | - |  |
| 29 | IC111919 | - | - |  |
| 30 | IC128179 | - | - |  |
| 31 | IC128457 | - | - |  |
| 32 | IC128507 | - | - |  |
| 33 | IC128520 | Kanpur | Uttar Pradesh |  |
| 34 | IC128521 | Kanpur | Uttar Pradesh |  |
| 35 | IC128524 | Kanpur | Uttar Pradesh |  |
| 36 | IC128525 | Kanpur | Uttar Pradesh |  |
| 37 | IC128526 | Kanpur | Uttar Pradesh |  |
| 38 | IC128553 | Kanpur | Uttar Pradesh |  |
| 39 | IC128555 | Kanpur | Uttar Pradesh |  |
| 40 | IC128587 | Kanpur | Uttar Pradesh |  |
| 41 | IC128590 | Kanpur | Uttar Pradesh |  |
| 42 | IC128592 | Kanpur | Uttar Pradesh |  |
| 43 | IC128594 | Kanpur | Uttar Pradesh |  |
| 44 | IC128619 | Kanpur | Uttar Pradesh |  |
| 45 | IC128624 | Kanpur | Uttar Pradesh |  |
| 46 | IC128629 | Kanpur | Uttar Pradesh |  |
| 47 | IC128631 | Kanpur | Uttar Pradesh |  |
| 48 | IC128637 | Kanpur | Uttar Pradesh |  |
| 49 | IC128638 | Kanpur | Uttar Pradesh |  |
| 50 | IC128650 | Kanpur | Uttar Pradesh |  |
| 51 | IC128652 | Kanpur | Uttar Pradesh |  |
| 52 | IC128654 | Kanpur | Uttar Pradesh |  |
| 53 | IC128656 | Kanpur | Uttar Pradesh |  |
| 54 | IC128692 | Almora | Uttar Pradesh |  |
| 55 | IC138364 | Pune | Maharashtra |  |
| 56 | IC138426 | Kanpur | Uttar Pradesh |  |
| 57 | IC138479 | Pune | Maharashtra |  |
| 58 | IC138521 | Almora | Uttarakhand |  |
| 59 | IC138524 | Almora | Uttarakhand |  |
| 60 | IC145331 | - | - |  |
| 61 | IC145602 | - | - |  |
| 62 | IC145780 | - | - |  |
| 63 | IC145808 | - | - |  |
| 64 | IC145811 | - | - |  |
| 65 | IC145882 | - | - |  |
| 66 | IC145884 | - | - |  |
| 67 | IC145916 | - | - |  |
| 68 | IC145951 | - | Uttar Pradesh |  |
| 69 | IC145977 | - | Uttar Pradesh |  |
| 70 | IC252370 | - | Uttar Pradesh |  |
| 71 | IC252392 | - | Uttar Pradesh |  |
| 72 | IC252431 | - | West Bengal |  |
| 73 | IC252432 | - | West Bengal |  |
| 74 | IC252433 | - | West Bengal |  |
| 75 | IC252439 | - | West Bengal |  |
| 76 | IC252441 | - | West Bengal |  |
| 77 | IC252443 | - | West Bengal |  |
| 78 | IC252444 | - | West Bengal |  |
| 79 | IC252445 | - | West Bengal |  |
| 80 | IC252448 | - | West Bengal |  |
| 81 | IC252450 | - | West Bengal |  |
| 82 | IC252453 | - | West Bengal |  |
| 83 | IC252455 | - | West Bengal |  |
| 84 | IC252456 | - | West Bengal |  |
| 85 | IC252457 | - | West Bengal |  |
| 86 | IC252458 | - | West Bengal |  |
| 87 | IC252459 | - | West Bengal |  |
| 88 | IC252469 | - | West Bengal |  |
| 89 | IC252472 | - | West Bengal |  |
| 90 | IC252490 | - | West Bengal |  |
| 91 | IC252497 | - | West Bengal |  |
| 92 | IC252499 | - | West Bengal |  |
| 93 | IC252520 | - | West Bengal |  |
| 94 | IC252541 | - | - |  |
| 95 | IC252542 | - | - |  |
| 96 | IC252547 | - | - |  |
| 97 | IC252591 | - | - |  |
| 98 | IC252611 | - | - |  |
| 99 | IC252629 | - | - |  |
| 100 | IC252650 | - | - |  |
| 101 | IC252673 | - | - |  |
| 102 | IC252676 | - | - |  |
| 103 | IC252684 | - | - |  |
| 104 | IC252686 | - | - |  |
| 105 | IC252706 | - | - |  |
| 106 | IC252723 | - | - |  |
| 107 | IC252725 | - | - |  |
| 108 | IC252767 | - | - |  |
| 109 | IC252768 | - | - |  |
| 110 | IC252818 | - | - |  |
| 111 | IC252819 | - | - |  |
| 112 | IC252892 | - | - |  |
| 113 | IC252983 | - | - |  |
| 114 | IC260877 | Chamoli | Uttar Pradesh |  |
| 115 | IC279320 | Shimla | Himachal Pradesh |  |
| 116 | IC279321 | Shimla | Himachal Pradesh |  |
| 117 | IC279333 | Shimla | Himachal Pradesh |  |
| 118 | IC279875 | Chamba | Himachal Pradesh |  |
| 119 | IC281566 | Tehri | Uttarakhand |  |
| 120 | IC290022 | - | - |  |
| 121 | IC290025 | - | - |  |
| 122 | IC290039 | - | - |  |
| 123 | IC290046 | - | - |  |
| 124 | IC290057 | - | - |  |
| 125 | IC290058 | - | - |  |
| 126 | IC290087 | - | - |  |
| 127 | IC290098 | - | - |  |
| 128 | IC290150 | - | - |  |
| 129 | IC290154 | - | - |  |
| 130 | IC290157 | - | - |  |
| 131 | IC290162 | - | - |  |
| 132 | IC290168 | - | - |  |
| 133 | IC290173 | - | - |  |
| 134 | IC290175 | - | - |  |
| 135 | IC290176 | - | - |  |
| 136 | IC290177 | - | - |  |
| 137 | IC290178 | - | - |  |
| 138 | IC290182 | - | - |  |
| 139 | IC290184 | - | - |  |
| 140 | IC290186 | - | - |  |
| 141 | IC290190 | - | - |  |
| 142 | IC290197 | - | - |  |
| 143 | IC290208 | - | - |  |
| 144 | IC290215 | - | - |  |
| 145 | IC290217 | - | - |  |
| 146 | IC290222 | Shimla | Himachal Pradesh |  |
| 147 | IC290226 | Shimla | Himachal Pradesh |  |
| 148 | IC290227 | Shimla | Himachal Pradesh |  |
| 149 | IC290231 | - | - |  |
| 150 | IC290241 | - | - |  |
| 151 | IC290242 | - | - |  |
| 152 | IC290243 | - | - |  |
| 153 | IC290244 | - | - |  |
| 154 | IC290258 | - | - |  |
| 155 | IC290261 | Shimla | Himachal Pradesh |  |
| 156 | IC290262 | Shimla | Himachal Pradesh |  |
| 157 | IC290264 | - | - |  |
| 158 | IC290280 | - | - |  |
| 159 | IC290281 | - | - |  |
| 160 | IC290298 | - | - |  |
| 161 | IC290299 | - | - |  |
| 162 | IC290302 | - | - |  |
| 163 | IC290305 | - | - |  |
| 164 | IC290309 | - | - |  |
| 165 | IC290311 | - | - |  |
| 166 | IC290314 | - | - |  |
| 167 | IC290316 | - | - |  |
| 168 | IC290325 | - | - |  |
| 169 | IC290326 | - | - |  |
| 170 | IC290327 | - | - |  |
| 171 | IC290329 | - | - |  |
| 172 | IC290342 | - | - |  |
| 173 | IC290665 | - | - |  |
| 174 | IC310100 | - | - |  |
| 175 | IC310120 | - | - |  |
| 176 | IC310124 | - | - |  |
| 177 | IC316100 | - | - |  |
| 178 | IC321153 | Sirmour | Himachal Pradesh |  |
| 179 | IC335670 | - | - |  |
| 180 | IC335671 | - | - |  |
| 181 | IC335683 | - | - |  |
| 182 | IC335704 | - | - |  |
| 183 | IC416080 | Ludhiana | Punjab |  |
| 184 | IC416082 | Ludhiana | Punjab |  |
| 185 | IC416083 | Ludhiana | Punjab |  |
| 186 | IC416084 | Ludhiana | Punjab |  |
| 187 | IC416092 | Ludhiana | Punjab |  |
| 188 | IC416094 | Ludhiana | Punjab |  |
| 189 | IC416281 | Ludhiana | Punjab |  |
| 190 | IC427210 | Una | Himachal Pradesh |  |

*No information available
