# Supplementary material for: Mining of Indian wheat germplasm collection for adult plant resistance to leaf rust
Source: PLoS One. 2019 Mar 28;14(3):e0213468. doi: 10.1371/journal.pone.0213468 (PMC6438482; doi:10.1371/journal.pone.0213468)
Supplement: S1 Fig — Representative molecular profiling of 48 wheat genotypes with linked DNA markers (a) CsLv34 (Lr34) and (b) Cfd71(Lr67) used for screening the known APR genes. (DOC) [file pone.0213468.s003.doc]

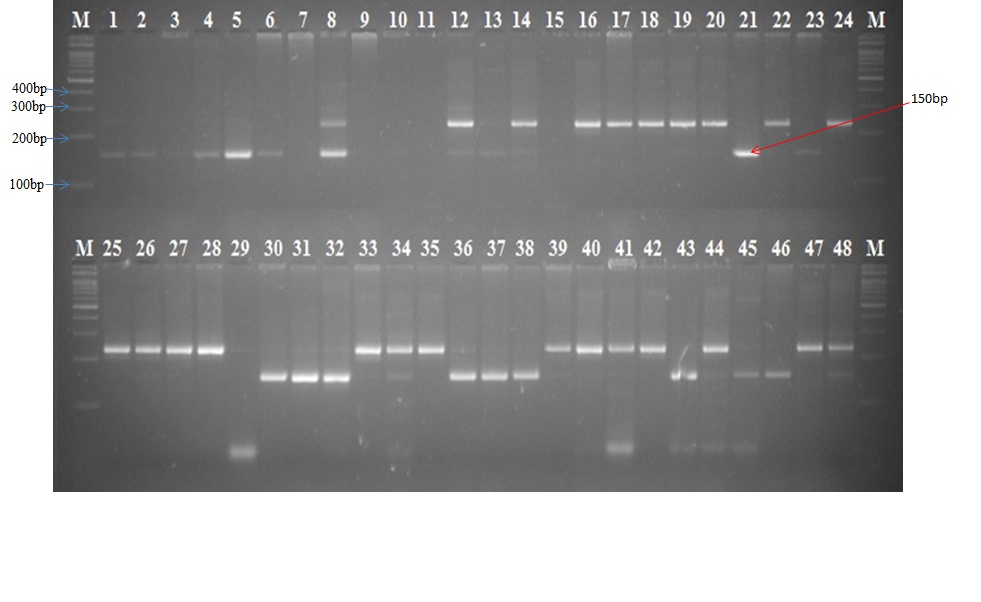


(a)


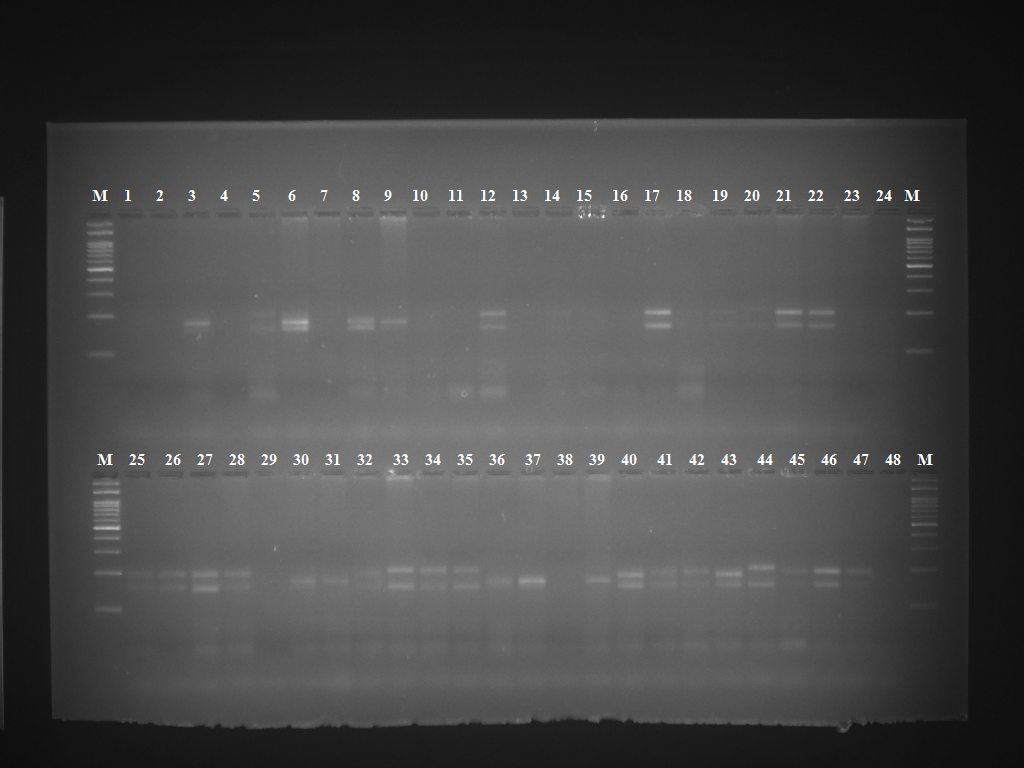


214 bp

200bp

100bp

300bp

400bp

**(b)**

**S1 Fig** Representative molecular profiling of 48 wheat genotypes with linked DNA markers (a) *CsLv34 (Lr34) and (b) Cfd71(Lr67)* used for screening the known APR genes.
